# Mate-value and relationship satisfaction: The moderating roles of mate retention behaviors

**DOI:** 10.1371/journal.pone.0262154

**Published:** 2022-01-18

**Authors:** Ali Babaeizad, Reza Fallahchai, Tayebeh Abbasnejad

**Affiliations:** 1 Department of Psychology, University of Hormozgan, Bandar Abbas, Iran; 2 Faculty of Management and Accounting, University of Hormozgan, Bandar Abbas, Iran; University of Padova, ITALY

## Abstract

Previous research indicates that mate retention strategies are associated with mate value and affect relationship satisfaction. The current research aimed to replicate previous findings in a non-WEIRD society (Iran) and to extend this research by investigating the moderating roles of individual and coalitional mate retention. Participants (n = 754; 416 women) in a committed, heterosexual relationship from two independent samples reported (1) their relationship satisfaction, (2) their partner’s mate value, (3) the frequency of performing individual mate retention, and (4) the frequency of requesting coalitional mate retention. Results indicated that there were positive associations between mate value, individual and coalitional Benefit-Provisioning mate retention behaviors, and relationship satisfaction. We found negative associations between individual and coalitional Cost-Inflicting mate retention behaviors and relationship satisfaction. We found that mate retention moderated the relationship between mate value and relationship satisfaction. Limitations of the current study are noted, and future directions are discussed.

## Introduction

Humans possess adaptations to solve problems of survival and reproduction [1], and many strategies have been devised to maximize the desired achievement of both, including the formation and maintenance of long-term emotional relationships. Although long-term relationships provide many benefits [2], such relationships carry sex-specific adaptive problems when infidelity occurs. Furthermore, a partner’s infidelity is an important threat to romantic relationships [3], and is linked with an increase in family stress, violence, depression, and a decline in self-esteem [4, 5]. The consequence of infidelity in men is an increase in the risk of cuckoldry, whereas in women is an increase in the risk of resource diversion [6]. Due to the importance of romantic relationships and their psychological effects on individuals [7], and imposed costs of infidelity on both men and women (i.e., relationship dissolution; [8]), they invest a considerable amount of time and resources to maintain these relationships. To achieve this goal, people employ strategies to maintain their relationships to prevent their partner from leaving the relationship or being poached by other rivals [7]. These strategies are described as mate retention tactics [9] ranging ‘‘from vigilance to violence” [10]. Employing these strategies leads to minimizing the risk of partner infidelity and relationship dissolution [11].

One of the strategies that both sexes use to maintain a mate is individual mate retention [10]. Gender differences were found in the performance of mate retention tactics. For example, women are more likely to perform appearance enhancement than men (e.g., to make themselves more attractive to their partners) because men value partner attractiveness more than women [12]. Men are more likely to use direct violence against their rivals than women because women value more about their partner’s ability to provide physical protection than men [13]. Although these tactics are universal throughout human societies, their manifestation depends on cultural contexts [14].

Additionally, humans live in social groups, and also recruit allies to assist in mate retention (coalitional mate retention; [15, 16]). For example, individuals can say positive things about a friend—thereby increasing that friend’s mating value [17]. Mate retention (which include behaviors that are performed alone, as well as those performed within coalitions) include positive “benefit-provisioning” behaviors (e.g., speaking positively about a partner’s appearance) and negative “cost-inflicting” behaviors (e.g., preventing a partner from talking to friends of the opposite sex; [6]. Provisioning benefits to the partner increases their self-esteem and relationship satisfaction [18] and is a low-risk way to minimize partner infidelity or relationship breakdown [16].

Furthermore, research results have shown that mate retention strategies are associated with mate value and affect relationship satisfaction [19] because mate retention is a function of self-assessment of one’s own mate value and his/her partner’s mate value in both male and female [20, 21].“Mate value” is defined as the genetic or material assistance [22], and refers to one’s desirability in the mating market [23], that includes one’s physical traits (e.g., the lower waist-to-hip ratio in women, higher shoulder-to-hip ratio in men; [24]) and social attributes (e.g., social status, intelligence; [25]). Miner, Shackelford, and Starratt [20] indicated that the mate value of men, not women, specifically predicts a wide range of benefit providing and cost inflicting mate retention behaviors. The results of this study showed that men with low mate value, compared to men with high mate value, engage in more risky and harmful mate retention behaviors, and men with high mate value may engage in less risky, based on beneficial behaviors, partly because they have the resources to afford these behaviors (e.g., financial resources to provide gifts to a partner). In another study, women are more likely to perform mate retention strategies when they consider their partners to have higher mate value than themselves, and men are more likely to use mate retention tactics when they consider themselves and their partners to have similar mate value [19].

One’s partner’s mate value is the strongest predictor of relationship satisfaction in general [26]. Both men and women who perceived their partners to have a higher mate value reported a higher level of satisfaction with their relationship [27]. People who were higher in mate value than their partners reported a decline in relationship satisfaction when their partners were less desirable than their alternatives [28]. In general, fewer differences in the mate value of partners were associated with higher relationship satisfaction [19]. Also, Shackelford and Buss [9] have proposed that relationship satisfaction has evolved into a psychological tool for monitoring the costs and benefits of romantic relationships. The use of benefit-provisioning mate retention tactics is associated with higher relationship satisfaction [19] while using cost-inflicting strategies is correlated with lower satisfaction with their relationship [29]. Based on the results of these studies, we hypothesized that the extent to which individuals are satisfied with their relationship may depend on their mate retention behaviors.

Previous research on the relationships between mate retention, mate value, and relationship satisfaction focus primarily on Western, Educated, Industrialized, Rich, and Democratic (WEIRD) samples. The evolutionary recurrent problems associated with partner infidelity should transcend cultural differences. In recent years, researchers’ interest in studying in non-WEIRD communities has increased [26, 30, 31]. Cross-cultural research from different societies has shown that human social behavior is different in many ways, and these cultural differences are essentially psychological [32]. In this regard, research findings indicate that people from WEIRD societies are different from people in non-WEIRD societies, including an average tendency to more individualism, impersonally prosocial, and less tendency to conformity, obedience, and kinship [30, 32, 33].

According to the literature, most Western countries have a culture of Dignity, but the East (and especially Iran) has an honor culture [34]. Honor is a cultural mindset that highlighted protecting oneself and one’s family by reputation maintenance [35]. In honor cultures, cooperativeness or competitiveness of social interactions depends on whether one’s reputation is threatened. If honoring is not threatened, individuals may seek a reputation by being very polite, warm, and hospitable [36]. Cultures of honor place a high value on the respectable social status of the family [37]. A fundamental expectation in honor cultures rooted in a collectivist and patriarchal structure is that individual adhere strictly to gender roles [38]. This social expectation dictates that women show their worth by showing loyalty, humility, unconditional compliance, and submission to their male relatives [39].

Iran has a "culture of honor" [40]. Iran is one of the largest countries in the Middle East with a population of about 85 million people [41]. According to official reports, the majority of whom (89%) consider themselves Muslims [41], with strong honor norms and there may be honor concerns [42, 43]. Iran is geographically and historically close to countries such as Pakistan, Egypt, Yemen, Azerbaijan, and Armenia, but is more educated and developed than these countries [44]. For example, in terms of education, at the national level, according to official reports [45], 93% of the population and 97% of young adults are literate (close to the WEIRD population). As the capital, Tehran is the largest city in Iran and is one of the most populous cities in West Asia. Its social and cultural structure is diverse and arises from the fusion of tradition and modernity. Iranian culture allows men and women to support socially acceptable behaviors to protect their partners as well as their family members [46].

For example, “Qeirat”, which is a special adjective for men in Persian, means protection against unwanted attention to his love partner [47]. Also, the equivalent of “Qeirat” in women is female jealousy in Persian. But jealousy in Iran seems a little different from women in individualistic cultures. The collective nature of Iranian culture allows Iranian women to engage their family members when they realize that their partners may be unfaithful. For example, if a woman feels that the relationship is in danger, it is traditionally acceptable to seek help from her husband’s family members [46]. In this regard, Shackleford [48] suggests overt behaviors that are defined as indicators of a culture of honor that these behaviors may be the product of psychological mechanisms that have evolved in response to repetitive evolutionary problems in mate retention. Studies in Iran indicated sex differences in mate retention behaviors and men performed higher mate retention behaviors [46, 49, 50]. In a study in Iran, Karimi-Malekabadi and Esmaeilinasab [51] found an association between self-promotion intrasexual rivalry and benefit-provisioning mate retention behaviors and reported that competitor-derogation attitudes in intrasexual competition are associated with cost-inflicting mate retention behaviors.

**Fig 1:**
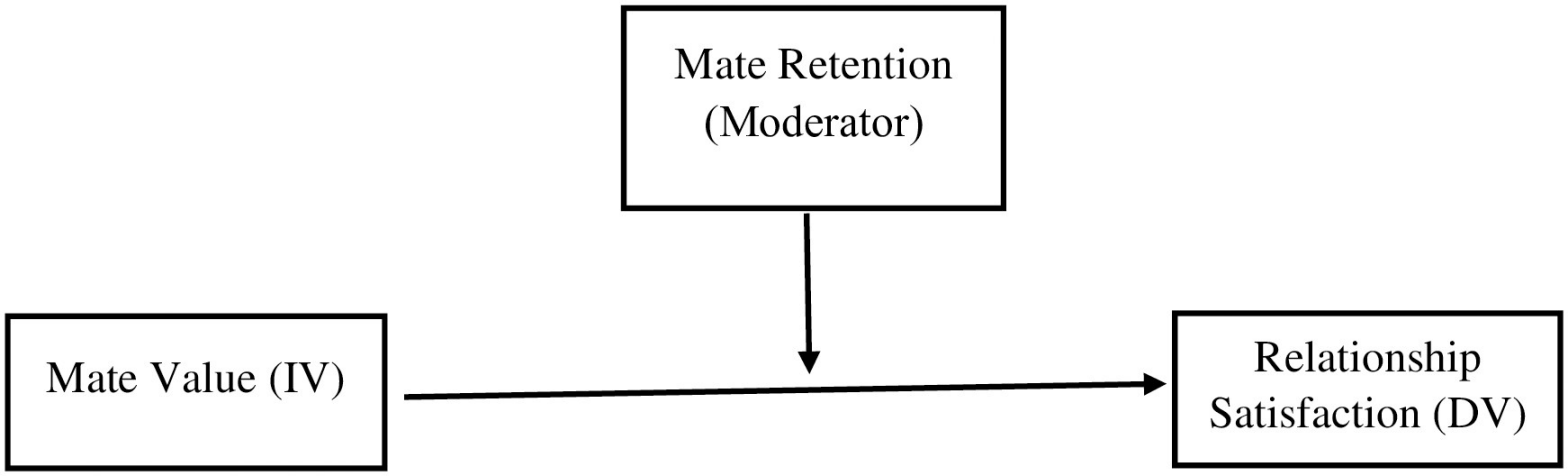
Moderation models testing the association between mate value and relationship satisfaction via mate retention behaviors.

Given that previous research on the associations between mate value, mate retention behaviors, and relationship satisfaction are conducted in WEIRD samples [19], the current study tries to expand the literature regarding these relationships in a non-WEIRD sample. The present study differs from prior studies in utilizing the relational mate value scale which measures individuals’ perceptions or judgments about their relationship quality as well as desirable traits. Prior research studies these variables and their associations separately and to our knowledge, only one study [19] investigated the association between mate value and relationship satisfaction and the mediation role of individual mate retention behaviors. The current study seeks to add to our understanding of the possible moderation role of both individual and coalitional mate retention in the association between mate value and relationship satisfaction. These unique mating aspects of Iran provide a unique test of hypotheses regarding mate retention. Following previous research, we hypothesize the following: Mate value will be positively associated with relationship satisfaction (H1), cost-Inflicting behavior will be negatively associated with relationship satisfaction while Benefit-Provisioning behavior will be positively associated with relationship satisfaction, (H2). Further, mate retention behaviors will be possible moderators in the relationship between mate value and relationship satisfaction (H3, see Fig 1).

## Method

### Participants

Study participants consisted of 754 (416 women) from two independent samples: 342 students recruited from universities of Tehran (190 women and 152 men) and 412 non-student community members (226 women and 186 men) selected from educational centers such as language institutes, institutes of science and technology studies, and the counseling centers that hold training programs for couples) and public places (such as Daneshjoo Park, Kourush Mall, Iran Mall, and so on) in the city of Tehran. The participants were heterosexual individuals between 19 and 40 years old and were in a committed relationship for at least five months. The mean age of all participants was 27.1 years old (SD = 8.75), (women: M = 25.78, SD = 8.33; men: M = 28.19, SD = 8.42). The average relationship length was 7.67 years (SD = 6.82). In terms of educational levels, 63.4% had a bachelor’s degree, 27.2% had an associate of arts degree and the rest had a high school diploma.

### Procedures

This research was approved by the Ethical Board of Hormozgan University of Medical Sciences (approval no.IR.HUMS.REC.1400.036) and conducted in accordance with the Declaration of Helsinki and its revised versions. All participants were informed about the study, fully understood the study purpose, and all provided written informed consent.

Participation was voluntary. We invited prospective participants via convenience sampling to take part in the study. Eligible participants who expressed interest in participating in this study completed a paper-and-pencil questionnaire package that included demographic information (including age, duration of their relationship, and other related information.), mate value scale, mate retention questionnaires (Individual and Coalitional), and the relationship satisfaction scale.

## Materials

### Mate value

To measure the mate value, the relational mate value scale developed by Eastwick & Hunt [52] and translated and adapted into Persian by Babaeizad, Fallahchai, and Abbasnejad [53] was used. This 14-item scale asks participants to report on a 7-point scale: 1 (*strongly disagree*) to 7 (*strongly agree*). Previous research by Eastwick & Hunt showed adequate internal consistency of the scale [52]. The results of Babaeizad et al. indicated that a coefficient alpha value of .91 for the entire questionnaire. Cronbach’s alpha of .88 was found in the current study.

### Individual mate retention

In order to assess mate retention behaviors, participants completed the Mate Retention Inventory-Short Form, (MRI-SF) [6]; translated and adapted to Persian by Atari, Barbaro, Shackelford, & Chegeni [46], which is a 38-item measure assessing performance frequencies of mate retention behaviors. The Persian version of the MRI-SF consists of a two-factor structure, highly agreeable with previous reports [14, 20], and demonstrates adequate internal consistency of mate retention tactics [46]. Participants were asked to report how often they performed each mate retention behavior using a 4-point scale (0 = *never*, 1 = *rarely*, 2 = *sometimes*, 3 = *often*). Following Atari et al., we constructed composite scores for the higher-order domains of Benefit-Provisioning and Cost-Inflicting mate retention behaviors. Internal consistency reliability values for the Benefit-Provisioning subscale, α = .83, and Cost-Inflicting subscale, α = .88, fell within an acceptable range.

### Coalitional mate retention

The Coalitional Mate Retention Inventory (CMRI; Pham, Barbaro, Mogilski, & Shackelford [16]; translated and adapted to Persian by Fallahchai, Babaeizad, Pham, & Eftekharian [50]. The questionnaire was designed to measure performance frequencies of specific behaviors of CMR behaviors [15]. The questionnaire has 44 items and was comprised of seven CMR tactics on a four-point scale (0 = *never*; 3 = *often*): manipulation, praise, vigilance, monopolizing time, therapy, gifts, and violence [15]. We calculated the scores for Benefit-Provisioning mate retention by averaging participants’ responses to subscales of Praise, Therapy, and Gifts. The scores for Cost-Inflicting mate retention comprise different subscales (e.g., Manipulation, Vigilance, Monopolizing Time, and Violence). The Cronbach’s alpha coefficient for Benefit-Provisioning was .78 and Cost-Inflicting was .81 in the current study.

### Relationship satisfaction

The Relationship Assessment Scale (RAS) [54], adapted to Persian by Dehshiri et al. [55] is a 7-item self-reported inventory designed to measure relationship satisfaction within a given relationship [54]. Items are scored on a 5-point Likert-type scale, ranging from 1 (*low satisfaction*) to 5 (*high satisfaction*). The RAS has previously demonstrated adequate validity [54, 56, 57] and adequate reliability [58, 59]. Results indicated that the Persian version of RAS has acceptable validity and reliability for an Iranian population [55]. The Cronbach’s alpha coefficient was .89 in the current study.

## Data analysis

The Statistical Package for Social Sciences (IBM SPSS, version 24.0) was used. Bivariate correlations for all variables were calculated. Gender differences in study variables were examined by performing independent t-tests.

To assess the associations between the independent variable (mate value) and the outcome variables (relationship satisfaction) and potential moderating variables (Cost-Inflicting and Benefit-Provisioning behaviors), hierarchical multiple regressions analyses were conducted. In the first model, in step one, age was entered. In the second step, all predictor variables (mate value, IMR and CMR Cost-Inflicting, and IMR and CMR Benefit-Provisioning behaviors) were entered. To test the moderation hypotheses, as recommended by Aiken and West [60], all continuous variables were mean-centered. We conducted four hierarchical multiple regressions analyses. In the first step, mate value and one of the mate retention behaviors were entered. In step 2, the interaction was examined. Separate models were conducted for men and women.

## Results

### Descriptive statistics

First, skewness, and kurtosis values were calculated to examine the normality characteristics of the variables. Results showed that all study variables were normally distributed in this sample. Levene’s Tests was calculated for homogeneity of variance. The results indicated that non-significant p values were found (all *p ˃* 0.050*)*, thus the assumption of the heterogeneity of variance was not violated. Then, independent t-tests were performed to test gender differences. As shown in Table 1, significant gender differences in IMR and CMR Cost-Inflicting, IMR and CMR Benefit-Provisioning, and relationship satisfaction were found. Men reported significantly higher scores in IMR Cost-Inflicting (t (753) = 2.37, *p* = 0.012), IMR Benefit-Provisioning (t (753) = 1.82, *p* = 0.051), CMR Cost-Inflicting (t (753) = 1.98, *p* = 0.019), CMR Benefit-Provisioning (t (753) = 1.87, *p* = 0.038), and relationship satisfaction (t (753) = 2.17, *p* = 0.023). No gender differences were found in age (t (753) = .78, *p* = 0.354), partner’s age (t (753) = -0.92, *p* = 0.212), Relationship length (t (753) = 0.62, *p* = 0.468), and mate value (t (753) = -0.81, *p* = 0.329).

**Table 1:** Descriptive of study variables.

Variable	Men		Women		df	t	p	Cohen’ d
	(N = 338)		(N = 416)					
	M	SD	M	SD				
**Age**	32.64	6.98	30.74	6.41	722	0.78	0.354	0.283
**Partner’s age**	29.81	6.22	32.17	6.83	727	-0.92	0.212	0.361
**Relationship length**	130.54	99.72	123.63	89.21	714	0.62	0.468	0.073
**Mate value**	7.06	1.07	7.21	1.51	742	-0.81	0.329	0.114
**IMR Cost-Inflicting**	1.42	0.53	.89	0.44	751	2.37	0.012	1.088
**IMR Benefit-Provisioning**	1.56	0.47	1.17	0.64	738	1.82	0.051	0.694
**CMR Cost-Inflicting**	1.19	0.63	.78	0.59	747	1.98	0.019	0.671
**CMR Benefit-Provisioning**	1.31	0.55	.96	0.57	729	1.87	0.031	0.624
**Relationship satisfaction**	3.94	0.58	3.53	0.86	741	2.17	0.023	0.558

Bivariate correlations between study variables to identify possible demographic control variables. Among demographic variables (e.g., age, partner’s age, and relationship length) previously found to be associated with study variables, results of bivariate correlations demonstrated that only age was correlated with study variables. Partial correlations among the target variables, controlling for age are reported in Table 2 for men (above the diagonal), for women (below the diagonal). Significant correlations among the target variables ranged from -0.32 to 0.51 for both men and women. Mate value was positively correlated with IMR and CMR Benefit-Provisioning and relationship satisfaction while it was negatively correlated with IMR and CMR Cost-Inflicting for both men and women. Positive correlations were found between IMR and CMR Cost-Inflicting and IMR and CMR Benefit-Provisioning for both men and women (see Table 2).

**Table 2:** Partial correlations between study variables controlling for age.

	**1**	**2**	**3**	**4**	**5**	**6**
**1. Mate value**	1	-0.28**	0.31**	-0.21*	0.18*	0.49**
**2. IMR Cost-Inflicting**	-0.31 **	1	0.45**	0.47**	0.39**	-0.27 **
**3. IMR Benefit-Provisioning**	0.30**	0.43**	1	0.47**	0.41**	0.30**
**4. CMR Cost-Inflicting**	-0.23*	0.48**	0.45**	1	0.43**	0.24*
**5. CMR Benefit-Provisioning**	0.20*	0.37**	0.51**	0.41**	1	0.23*
**6. Relationship satisfaction**	0.45**	-0.32**	0.33**	0.21*	0.17*	1

Note.

* p ≤ .05,

** p ≤ .01

### Associations between mate value, mate retention, and relationship satisfaction

The hierarchical regression analysis was conducted to determine the associations between the predictor variables (mate value and mate retention behaviors) and the outcome variable (relationship satisfaction) after controlling age, for women and men separately. Age was entered in step 1 and mate value and mate retention behaviors were entered in step 2.

In men, age accounted for 1.2% of the variation in relationship satisfaction, F (1, 336) = 5.40, *p* = .042). In step 2, the predictor variables were entered together and explained an additional 63% of the variation in relationship satisfaction and significantly improved the model, F (5, 331) = 97.86, *p* < .0001. As it has been shown in Table 3, mate value had a positive and significant association with relationship satisfaction with (*β* = 0.62, *p* <0.000). A significant negative association between IMR Cost-Inflicting and relationship satisfaction were found (*β* = -0.15, *p* = 0.001) whereas IMR Benefit-Provisioning was positively associated with relationship satisfaction (*β* = 0.21, *p* = 0.001). We did not find significant association between CMR Cost-Inflicting and relationship satisfaction (*β* = -0.06, *p* = 0.097) and CMR Benefit-Provisioning and relationship satisfaction (*β* = 0.05, *p* = 0.217).

**Table 3:** Hierarchical regression analyses for variables predicting relationship satisfaction, for men and women.

Variable	Men		Women	
	Step 1	Step 2	Step 1	Step 2
	β	β	β	β
**Age**	-.12	-.01	-.13	-.02
**Mate Value**		.62***		.68***
**IMR Cost-Inflicting**		-.15**		-.18**
**IMR Benefit-Provisioning**		.21**		.24***
**CMR Cost-Inflicting**		-.06		-.09
**CMR Benefit-Provisioning**		.05		.07
**Δ*R*** ^***2***^	.012	.63	.013	.67
***R*** ^***2***^	.012	.642	.013	.683
**F**	5.40*	97.86***	6.15***	168.42***

Notes:

**p < .01,

*** p < .0001;

standardized regression coefficients are reported.

In women, age accounted for 1.3% of the variation in relationship satisfaction, F (1, 414) = 6.15, *p* = .021). In step 2, the predictor variables were entered together and explained an additional 67% of the variation in relationship satisfaction and significantly improved the model, F (5, 409) = 168.42, *p* < .0001. Mate value had a positive and significant association with relationship satisfaction with (*β* = 0.68, *p* <0.000) (see Table 3). A significant negative association between IMR Cost-Inflicting and relationship satisfaction were found (*β* = -0.18, *p* = 0.001) whereas IMR Benefit-Provisioning was positively associated with relationship satisfaction (*β* = 0.24, *p* = 0.001). We did not find significant association between CMR Cost-Inflicting and relationship satisfaction (*β* = -0.09, *p* = 0.072) and CMR Benefit-Provisioning and relationship satisfaction (*β* = 0.07, *p* = 0.183).

### H3. Moderation role of mate retention

Tables 4 and 5 reported the results of hierarchical regression analyses of moderating roles of IMR Cost-Inflicting and IMR Benefit-Provisioning in the association between mate value and relationship satisfaction.

**Table 4:** Results of moderation effect of IMR cost-inflicting on the relationship between mate value and relationship satisfaction.

Variable	Men		Women	
	Step 1	Step 2	Step 1	Step 2
	β	β	β	β
**Mate Value**	.40***	.36***	.43***	-.39***
**IMR Cost-Inflicting**	-.36***	-.31***	-.39***	-.33***
**Mate Value × IMR Cost-Inflicting**		.28**		.30***
**Δ*R*** ^***2***^	.61	.05	.64	.07
***R*** ^***2***^	.61	.66	.64	.71
**F**	231.41***	178.72***	327.89***	209.47***

Notes:

**p < .01,

*** p < .0001;

standardized regression coefficients are reported.

**Table 5:** Results of moderation effect IMR benefit-provisioning on the relationship between mate value and relationship satisfaction.

Variable	Men		Women	
	Step 1	Step 2	Step 1	Step 2
	β	β	β	β
**Mate Value**	.45***	.31***	.48***	.34***
**IMR Benefit-Provisioning**	.33***	.23**	.37***	.25**
**Mate Value × IMR Benefit-Provisioning**		.34**		.39***
**Δ*R*** ^***2***^	.63	.06	.67	.08
***R*** ^***2***^	.63	.69	.67	.75
**F**	378.33***	243.98***	395.85***	286.91***

Notes:

**p < .01,

*** p < .0001;

standardized regression coefficients are reported.

#### IMR Cost-Inflicting

In men, mate value and IMR Cost-Inflicting were entered in step one. They significantly contributed to the regression model, *F* (2, 335) = 231.41, *p*< .0001) and accounted for %61 of the variation in relationship satisfaction. In step 2, the interactions between mate value and IMR Cost-Inflicting accounted for %5 of the variance in relationship satisfaction scores *F* (3, 334) = 178.72, *p*< .0001). In women, mate value and IMR Cost-Inflicting entered in step one and significantly contributed to the regression model, *F* (2, 413) = 327.89, *p*< .0001). They accounted for %64 of the variation in relationship satisfaction. In step 2, the interactions between mate value and IMR Cost-Inflicting accounted for %7 of the variance in relationship satisfaction scores *F* (3, 412) = 209.47, *p*< .0001).

#### IMR Benefit-Provisioning

In men, mate value and IMR Benefit-Provisioning entered in step one and significantly contributed to the regression model, *F* (2, 335) = 378.33, *p*< .0001) and accounted for %63 of the variation in relationship satisfaction. In step 2, the interactions between mate value and IMR Benefit-Provisioning accounted for %6 of the variance in relationship satisfaction scores *F* (3, 334) = 243.98, *p*< .0001). In women, mate value and IMR Benefit-Provisioning entered in step one and significantly contributed to the regression model, *F* (2, 413) = 395.85, *p*< .0001) and accounted for %67 of the variation in relationship satisfaction. In step 2, the interactions between mate value and IMR Benefit-Provisioning accounted for %8 of the variance in relationship satisfaction scores *F* (3, 412) = 286.91, *p*< .0001).

## Discussion

We found that mate value and relationship satisfaction were positively associated (H1), relationship satisfaction was positively associated with benefit-provisioning mate retention, and negatively associated with cost-inflicting mate retention (H2). We also found that mate retention moderated the relationship between mate value and relationship satisfaction, suggesting that a more complete understanding of the adaptive problems (and solutions) associated with long-term mating may require a model that includes all the target variables included in the current research.

Our overall results from Iranian samples are remarkably similar to previous research that used WEIRD samples.

The first finding of this study indicates a significant positive relationship between mate value and relationship satisfaction for both women and men. This finding is somewhat consistent with the findings of Nowak and Danel [61] who reported that relationship satisfaction is positively associated with women’s perception of their partners’ mate value. Research results show that mate value discrepancy affects relationship satisfaction, communication commitment, and mate retention behaviors [26, 62]. For example, research by Nowak and Danel [61] has shown that women’s evaluations of their partners’ mate value play a more important role in relationship satisfaction than their own mate value. In addition, research has shown that couples who find their partner to have a higher mate value are more likely to adopt Benefit-Provisioning strategies. Applying Benefit-Provisioning strategies to the partner, in turn, increases the value of the individual and leads to increased satisfaction with the relationship [19].

The second finding of this study showed that both women and men reported the use of Benefit-Provisioning strategies more than Cost-Inflicting strategies in both individual and coalitional mate retention strategies. In explaining this finding, researchers believe that mate value acts as an evolutionary mechanism to enable individuals to invest their resources and time properly [63] and that mate retention is also a function of the performance of self-assessing mate value and evaluating one’s partner’s mate value in both men and women [20, 62]. Previous research has shown that when spouses believe that their partners have greater mate value than themselves, they tend to use more Benefit-Provisioning strategies [19]. Also, men with higher mate values are more likely to engage in Benefit-Provisioning mate retention behaviors than men with lower mate values [62]. In this regard, women report that men with higher mate values, from the perspective of mate retention strategies, use more Benefit-Provisioning behaviors (e.g., giving gifts, praising a partner, and holding hands) [62].

The third finding of this study indicated that the use of individual and coalitional mate retention strategies is higher in men than women. This finding regarding sex differences in mate retention behaviors is consistent with previous research [46, 49, 50], suggesting that men reported greater performance frequency of mate retention behaviors. Gender differences in the performance of mate retention behaviors indicate that men perform more Benefit-Provisioning and Cost-Inflicting strategies than women. This finding is consistent with the results of studies by Atari et al. [46], Barbaro et al. [64] in which participants reported greater Benefit-Provisioning mate retention behaviors in comparison with Cost-Inflicting, and a gender difference was reported in a US sample for Cost-Inflicting mate retention, rather than Benefit-Provisioning. Explaining our finding, it can be said that men make more efforts to maintain their attractive partners [65], and when they realize the high risk of sexual infidelity, they are more likely to use mate retention strategies than women [65, 66], as well as when their spouse shows signs of fertility such as physical attractiveness [9].

In addition, the performance of Benefit-Provisioning mate retention behaviors indicates an interest in a long-term committed relationship. According to previous research, these actions may be interpreted as providing Benefit-Provisioning behaviors rather than Cost-Inflicting behaviors [55]. Explaining this difference, Buss, and Shackelford [9] state that men who believe that their wives have higher mate value exhibit more mate retention behaviors. Also, this gender difference can usually be attributed to specific religious influences on the type of behavior in a romantic relationship [64]. On the other hand, the higher frequency of mate retention behavior in Iran by men compared to women can be considered a reflection of “Qeirat” in Iranian culture. For example, "if a potential competitor flirts a man’s partner or intends to have a relationship, it is socially acceptable for a man to react or use violence against a potential poacher" [46]. Such reactions are sometimes supported by the Islamic law that currently prevails in Iran [64].

The fourth finding of this study showed that relationship satisfaction is positively associated with Benefit-Provisioning mate retention and negatively associated with Cost-Inflicting mate retention in both women and men. This finding is consistent with the results of Salkicevic et al. [19]. Researchers believe that the use of Benefit-Provisioning mate retention strategies creates a positive relationship atmosphere in which both partners experience relationship satisfaction. Such behaviors give the couple reasons to stay and invest in their partner. It can also be said that if men use more common signs of possession, it means that they consider their partner more valuable and by this strategy, they want to show that the competition for possession of this woman is over, and she is no longer available. On the other hand, women value men more, when give them more positive incentives, men who provide them with material goods, which, of course, agrees with the fact that men are more valuable if they are the breadwinners of the house. In addition, we can say that when the relationship satisfaction increases, both partners use Benefit-Provisioning strategies more, and this, in turn, makes both more satisfied [19].

The last finding of this study showed that Benefit-Provisioning mate retention strategies played a moderating role in the relationship between mate value and relationship satisfaction in a way that was associated with increased relationship satisfaction. Benefit-Provisioning strategies are most commonly used by partners with higher mate value and /or for partners with higher mate value. Such behaviors give the couple reasons to stay and invest in their partner and create a positive relationship atmosphere in their relationship, which in turn increases satisfaction with the relationship. So, we can say that when the satisfaction of the relationship increases, both people in the relationship use Benefit-Provisioning strategies more, and in turn, using more Benefit-Provisioning strategies links with more satisfaction of both partners [19].

Another explanation for this finding is that the "ideal standards" model of relationship satisfaction (RS) predicts that RS will be negatively affected when partners’ perceptions of ideal standards are not met. The premise of this model is that natural selection prefers reduced RS, which motivates us to abandon relationships that do not meet our expectations of our desired mates [28, 67, 68] proposed an important modification of the model because abandoning the relationship would only be beneficial if it were reasonably possible to find a mate who met the individual’s standards.

## Limitations

Although this study is one of the few studies examining the associations between mate value and relationship satisfaction in an Iranian sample, it has some limitations. First, data for this study was based on cross-sectional data, which resulted in a moderation analysis without an experimentally manipulated variable, we cannot make causal inferences. Future longitudinal research may shed light on the relationship between mate value, mate retention behaviors, and relationship satisfaction. The second limitation of this study was that the data were obtained from men and women who were in a relationship or married, not from dyads. Therefore, in future studies, it is suggested that this study be performed with dyads. The third limitation of this study was that its data were collected from individuals who had either a short-term (non-marriage) or long-term (marriage) relationship. The study and comparison of these two groups should be done according to the studied variables.

## Supporting information

S1 Dataset (XLSX)
